# Chemodiversity of Arctic Plant *Dryas oxyodonta*: LC-MS Profile and Antioxidant Activity

**DOI:** 10.3390/plants13060868

**Published:** 2024-03-18

**Authors:** Nina I. Kashchenko, Daniil N. Olennikov, Nadezhda K. Chirikova

**Affiliations:** 1 Laboratory of Biomedical Research, Institute of General and Experimental Biology, Siberian Division, Russian Academy of Science, 6 Sakh’yanovoy Street, 670047 Ulan-Ude, Russia; olennikovdn@mail.ru; 2 Department of Biochemistry and Biotechnology, North-Eastern Federal University, 58 Belinsky Street, 677027 Yakutsk, Russia; hofnung@mail.ru

**Keywords:** Dryadoideae, Rosaceae, flavonols, sexangularetin, corniculatusin, quercetin, kaempferol, DPPH, ABTS

## Abstract

*Dryas oxyodonta* Yuz. is a perennial evergreen shrub from the Rosaceae family. *D. oxyodonta* thrives in subalpine and subarctic regions, as well as in highlands spanning from Central Asia to Siberia and Mongolia. Owing to a lack of information on its chemical composition, we conducted qualitative and quantitative chromatographic analyses on extracts from the leaves and flowers of *D. oxyodonta* sourced from various Siberian habitats. Employing high-performance liquid chromatography with photodiode-array detection and electrospray ionization triple-quadrupole mass spectrometric detection, we identified 40 compounds, encompassing gallotannins, hydroxycinnamates, procyanidins, catechins, flavonoids, and triterpenes. All Siberian populations of *D. oxyodonta* exhibited a notable abundance of phenolic compounds. Furthermore, we identified rare glycosides, such as sexangularetin and corniculatusin, as potential markers of the chemodiversity within the *Dryas* genus. Extracts from the flowers and leaves were effective scavengers of free radicals, including DPPH^•^, ABTS^•+−^, O_2_^•−^, and ^•^OH radicals. Our findings unequivocally establish *D. oxyodonta* as a rich source of phenolic compounds with potent antioxidant activity, suggesting its potential utility in developing novel functional products.

## 1. Introduction

The Rosaceae family is horticulturally important, containing various economically significant fruiting and ornamental species [1]. Chemotaxonomic investigations into Rosaceae have been conducted worldwide, examining botanical [2], genomic [3], and chemical perspectives [4]. Despite extensive research, debates persist regarding the classification of certain genera within the Rosaceae family. One such controversial genus is *Dryas*, which belongs to the subfamily Dryadoideae [5], although it has historically been classified as a separate family (Dryadaceae), tribe (Dryadeae), or subtribe (Dryadinae) [6]. Thriving predominantly in the cold regions of the Northern Hemisphere, particularly in subalpine and subarctic zones, the *Dryas* genus plays a significant role in the vegetation of high-mountain arctic and alpine tundras, often symbolizing these environments [7]. The ability of *Dryas* to form dense alpine thickets is likely attributable to the structure of its fruit—an achene characterized by a long, persistent, and feathery shaft. *Dryas* has limited distribution in snowy conditions because achenes fall very close to the parent plant [8].

**Figure 1:**
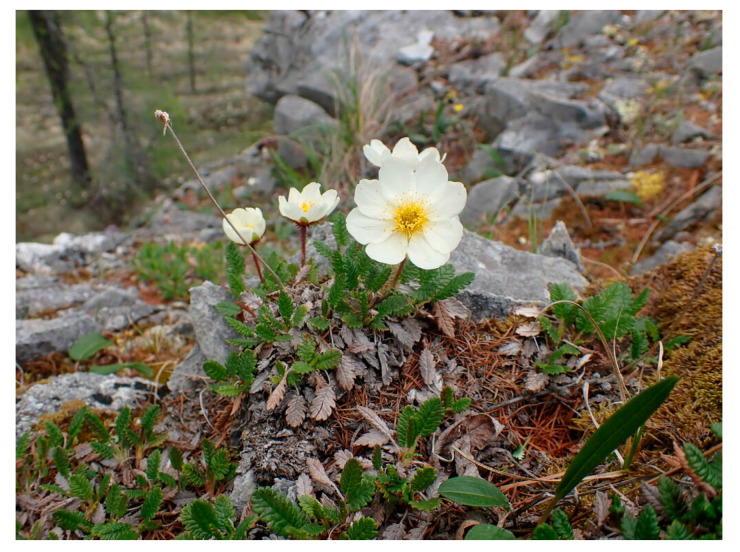
*Dryas oxyodonta* Juz. (Okinsky District, Buryatia Republic, Russia; CC BY-NC).

*Dryas oxyodonta* Juz. is a perennial evergreen shrub, forming cushion-shaped growths with creeping, branched stems reaching heights of up to 8 cm (Figure 1). Its leaves, simple and petiolate, have oblong blades measuring 1–3 cm in length. These leaves exhibit a two-toned appearance, with dark green tops and whitish, tomentose undersides adorned with blunt serrations along the edges. The solitary white flowers bloom on 3–6-centimeter-long peduncles [9]. *D. oxyodonta* is distributed in subalpine and subarctic regions and in highlands stretching from Central Asia to Siberia and Mongolia [10]. Among Siberian ethnic groups, particularly the Yakuts and Buryats, *D. oxyodonta* is used in traditional folk medicine for treating diarrhea and aiding digestion [11,12].

Despite its medicinal significance, the chemical composition of *D. oxyodonta* remains largely unexplored. Currently, there are no data regarding its chemical makeup. Among the *Dryas* genus, *D. octopetala* is the most studied species. It is known that the leaves of *D. octopetala*, collected in the mountains of France and Norway, contain procyanidin, propelargonidin, quercetin, kaempferol, isorhamnetin, corniculatusin, sexangularetin, limocitrin, and gossypetin [13]. Leaves of *D. octopetala*, collected in the mountain valleys of the Dolomites, yielded (+)-epicatechin and six flavonol glycosides: corniculatusin 3-*O*-arabinofuranoside, corniculatusin 3-*O*-galactopyranoside, sexangularetin 3-*O*-galactoside, hyperoside, avicularin, and guaijaverin [14]. In traditional medicine, the flowers and leaves of *D. octopetala* are used as digestive, antidiarrheal [14], cardiovascular, and neurological remedies [15].

The similarity in chemical composition and the identification of patterns in the metabolomes of plant species from the same family offer insights into their chemodiversity. Chemical compounds aid in species identification and quality control of herbal medicinal products that are increasing in popularity [16]. To perform chemodiversity studies, advanced analytical tools like high-performance liquid chromatography with photodiode-array detection and electrospray ionization triple-quadrupole mass spectrometric detection (HPLC–PDA–ESI–tQ–MS) are indispensable, serving as instrumental tools for fingerprinting plant extracts.

As part of our ongoing investigation into the metabolomes of Rosaceae family members [17,18,19], we conducted, for the first time, a comprehensive qualitative and quantitative chromatographic analysis of extracts from *D. oxyodonta*’s leaves and flowers, collected from diverse Siberian habitats. Employing HPLC–PDA–ESI–tQ–MS, we evaluated these extracts for their chemical constituents and antioxidant potential. Additionally, we identified specific chemotaxonomic markers characteristic of the *Dryas* genus.

## 2. Results and Discussion

### 2.1. Metabolome of Dryas oxyodonta

**Figure 2:**
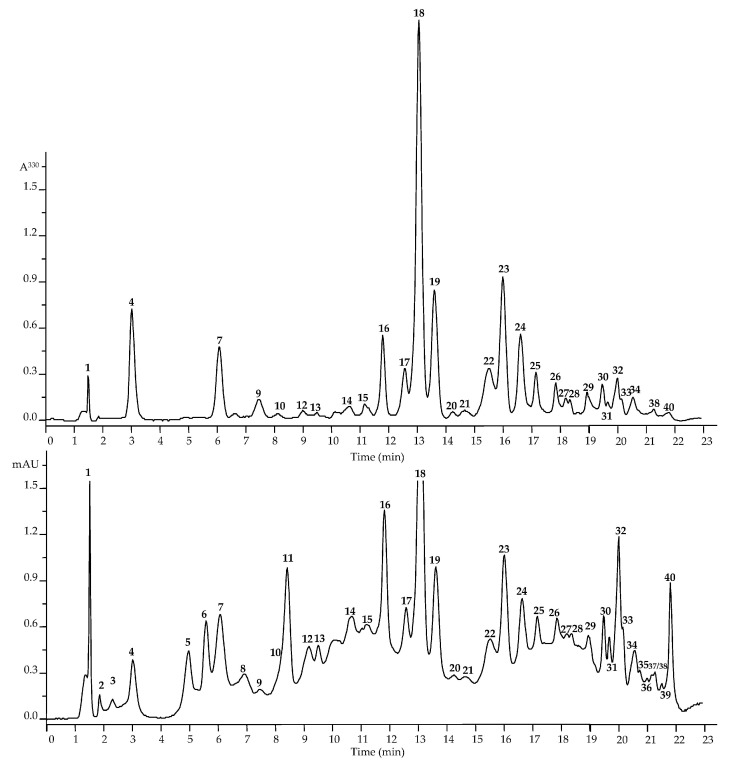
High-performance liquid chromatography data of *Dryas oxyodonta* extracts (top figure―HPLC-PDA chromatogram, λ 330 nm; bottom figure―HPLC-MS chromatogram, TIC, negative ionization). The numbering of the compounds is indicated as in Table 1.

The analysis of metabolites in *D. oxyodonta* extracts was conducted using the HPLC–PDA–ESI–tQ–MS methodology. This comprehensive analysis allowed us to identify 40 distinct compounds (Figure 2 and Figure S1; Table 1). The identification process adhered to the recommended minimum reporting standards for chemical analysis, as outlined by the Chemical Analysis Working Group. Specifically, compounds were identified using retention times, UV and MS spectra, and by comparison with standard compounds and the existing literature [20]. Metabolite identification was performed at two levels, with nineteen compounds fully characterized at the first level and twenty-one provisionally annotated at the second level.

#### 2.1.1. Gallotannins, Hydroxycinnamates, Procyanidins, and Catechins

The analysis of *D. oxyodonta* revealed representatives from six compound groups, encompassing gallotannins, hydroxycinnamates, procyanidins, catechins, flavonoids, and triterpenes. Notably, two gallotannins were discerned, each at varying levels of identification. The presence of 1-*O*-galloyl glucose (**3**), also known as glucogallin, was confirmed by comparing retention times and spectral characteristics with a reference standard. Additionally, the nature of galloyl glucose (**2**) was elucidated by comparing UV and MS data with the existing literature, particularly focusing on the deprotonated ion and the loss of particles with *m*/*z* 152 (a gallic acid fragment) [22].

Five hydroxycinnamates were identified in the *D. oxyodonta* extract through comparison with reference standards [21]. Among these, three derivatives of caffeic acid—6-*O*-caffeoyl glucose (**1**), 1-*O*-caffeoyl glucose (**4**), and 1,6-di-*O*-caffeoyl glucose (**10**)—were confirmed, along with derivatives of *p*-coumaric and ferulic acids, identified as 1-*O*-*p*-coumaroyl glucose (**7**) and 1-*O*-feruloyl glucose (**9**), respectively. Furthermore, two procyanidins, B1 (**5**) and B2 (**8**), along with two catechins, (+)-catechin (**6**) and (−)-epicatechin (**11**), were characterized at the first level of metabolite identification through comparison of retention times, UV spectra, and MS data with reference compounds [22,23]. Notably, procyanidin and (+)-epicatechin were previously detected in *D. octopetala* leaves [13,14].

#### 2.1.2. Flavonoids

Flavonoids were the predominant group of compounds in the *D. oxyodonta* extracts. The detected flavonol-*O*-glycosides, identified based on absorption spectra and deglycosylated fragment sizes, primarily belonged to derivatives of quercetin (**12**–**15**, **18**, **19**, **22**, **23**, **26**, **29**, **30**, **33**, and **34**; 254 ± 1, 265 ± 1, 352 ± 2 nm; 253 ± 1, 265 ± 1, and 333 ± 3 nm; aglycone with *m*/*z* 301), kaempferol (**24**, **25**, **38**, and **40**; 250 ± 3 nm, 350 ± 2 nm; 252 ± 3, and 332 ± 2 nm; aglycone with *m*/*z* 285), sexangularetin (**20**, **21**, **31**, and **32**; 270 ± 2, 356 ± 2 nm; 271 ± 2, and 335 ± 2 nm; aglycone with *m/z* 315), and corniculatusin (**16**, **17**, **27**, and **28**; 273 ± 2, 360 ± 2 nm; 273 ± 2, and 343 ± 3 nm; aglycone with *m*/*z* 331).

Four quercetin derivatives—hyperoside (**18**; 463 → 301), guaijaverin (**19**; 433 → 301), reynoutrin (**22**; 433 → 301), and avicularin (**23**; 433 → 301)—were identified through comparison of retention times, UV spectra, and MS data with reference compounds. Additionally, compounds **12** and **14**, displaying similar UV and MS characteristics to quercetin derivatives, yielded deprotonated ions [M − H]^−^ with *m*/*z* 787 and 625, respectively, along with specific daughter ions following the elimination of three and two *O*-bonded hexose moieties (*m/z* 787 → 625 → 463 → 301 and *m*/*z* 625 → 463 → 301, respectively) [24]. The tentative structures of compounds **12** and **14** were identified as quercetin 3-*O*-trihexoside and quercetin 3-*O*-dihexoside, respectively. Compounds **13** and **15** were also determined to be quercetin glycosides, containing pentose and hexose fragments within their structures. The presence of these fragments was further validated by a mass loss of 162 and 132 a.m.u., corresponding to hexose and pentose, respectively [25]. The proposed formulae of **13** and **15** were detected to be quercetin 3-*O*-pentoside-*O*-dihexoside and quercetin 3-*O*-pentoside-*O*-hexoside, respectively. Compounds **12**–**15** exhibited UV patterns that were consistent with flavonol glycosides (254 ± 1, 265 ± 1, and 352 ± 2 nm), while compounds **26**, **29**, **30**, **33**, and **34** displayed UV data characteristic of flavonol caffeoylglycosides (253 ± 1, 265 ± 1, and 333 ± 3 nm). The provisional structures of compounds **26**, **29**, **30**, **33**, and **34** were determined based on a specific hypsochromic shift of band I in the UV spectrum, along with the loss of the caffeoyl moiety (162 a.m.u.) in the mass spectrum. The assumed structures for compounds **26** and **30** were identified as quercetin *O*-caffeoyl-*O*-pentoside-*O*-hexoside and quercetin *O*-caffeoyl-*O*-pentoside, respectively. Compounds **30**, **33**, and **34** were characterized as quercetin *O*-caffeoyl-*O*-pentoside [31].

Two kaempferol derivatives—trifolin (kaempferol 3-*O*-galactoside, **24**; 447 → 285) and juglanin (kaempferol 3-*O*-arabinofuranoside, **25**; 417 → 285)—were characterized at the first level of identification through comparison of retention times, UV spectra, and MS data with reference compounds. The proposed structures of the acylated kaempferol derivatives were inferred from characteristic UV spectra (252 ± 3 and 332 ± 2 nm), deglycosylated fragments, and daughter ions formed upon the loss of caffeoyl moieties in the mass spectra, indicating kaempferol *O*-caffeoyl-*O*-hexoside and kaempferol *O*-caffeoyl-*O*-pentoside as tentative formulae for compounds **38** and **40**, respectively [30].

Derivatives **20**, **21**, **31**, and **32** were identified as containing either sexangularetin or 8-methoxykaempferol as an aglycone moiety, making them rare compounds. Tentatively identified non-acylated sexangularetins were observed to be glycosides with an aglycone daughter ion at *m*/*z* 315 and carbohydrate moieties as either hexose (sexangularetin *O*-hexoside, **20**; 477 → 315) or pentose (sexangularetin *O*-pentoside, **21**; 447 → 315) [29]. Compounds **20** and **21** are likely sexangularetin 3-*O*-galactoside and sexangularetin 3-*O*-arabinoside, respectively, because these compounds have been previously detected in the leaves of *D. octopetala* [14]. Acylated sexangularetin glycosides include proposed structures such as sexangularetin *O*-caffeoyl-*O*-hexoside (**31**) and sexangularetin *O*-caffeoyl-*O*-pentoside (**32**). These compounds showed specific bands in the UV spectrum (271 ± 2 and 335 ± 2 nm) and the loss of the caffeoyl moiety in the mass spectrum (*m*/*z* 639 → 477 and 609 → 447, respectively) [29].

Another group of rare flavonols in the *D. oxyodonta* extract includes corniculatusin or 8-methoxyquercetin derivatives. Non-acylated corniculatusin *O*-hexoside (**16**) and corniculatusin *O*-pentoside (**17**) were presumably identified after a comparison of UV and MS data with information from the literature [14]. Possible structures of **16** and **17** are corniculatusin 3-*O*-galactoside and corniculatusin 3-*O*-arabinoside, respectively, which were previously isolated from the leaves of *D. octopetala* [14]. Acylated corniculatusin glycosides demonstrated typical bands in the UV spectra (273 ± 2 and 343 ± 3 nm). Compound **27** (deprotonated ion at *m*/*z* 655) demonstrated a loss of caffeoyl and hexose (*m*/*z* 655 → 493, 331), which is characteristic of corniculatusin *O*-caffeoyl-*O*-hexoside. Compound **28** yielded an [M − H]^−^ ion with *m*/*z* 625 and two fragment ions, resulting from the loss of caffeoyl and pentose. The structure of **28** was determined to be corniculatusin *O*-caffeoyl-*O*-pentoside [14].

Previously, the flavonoids hyperoside, avicularin, and guaijaverin were identified in *D. octopetala* leaves [14]. It is also worth noting that the aglycones quercetin, kaempferol, corniculatusin, and sexangularetin were previously discovered in the same plant [13]. However, in the *D. oxyodonta* extract, we only identified flavonoid glycosides. Therefore, the compounds reynoutrin, trifolin, and juglanin were identified for the first time in the *Dryas* genus.

#### 2.1.3. Triterpenes

Four triterpenes were discerned in the *D. oxyodonta* extract, including tormentic (**35**), corosolic (**36**), and ursolic (**37**) acids. Their identification was achieved by comparing their UV spectra, MS data, and *t_r_* with those of reference compounds. The ursolic acid isomer (**39**) was provisionally annotated by comparing its UV and MS spectra with data from the literature. Notably, triterpenoids have not been previously reported in species of the *Dryas* genus.

Thus, this study’s metabolomic profile of the *D. oxyodonta* extract provides comprehensive insights into its chemical composition and unveils novel compounds within the *Dryas* genus.

### 2.2. Chemodiversity Significance of D. oxyodonta Metabolites for the Dryas Genus

We attempted to identify specific markers of chemodiversity for the *Dryas* genus to clarify its potential taxonomic position from a chemical perspective. Our investigation focused on several compound groups (gallotannins, hydroxycinnamates, procyanidins, catechins, triterpenes, and flavonoids), comparing them with existing data on *D. octopetala* and representatives from other subfamilies.

Gallotannins are widely distributed in representatives of the Rosaceae family [32]. Gallotannins, in particular glucogallin, are known to participate in the biosynthesis of 1,2,3,4,6-pentagalloylglucose, a precursor to ellagitannins [33] frequently found in the Rosoideae subfamily [19,34,35]. Notably, ellagitannins were not detected in *D. oxyodonta* despite the fact that the genus *Dryas* was previously included in the Rosoideae subfamily [36]. Given the widespread distribution of gallotannins in the Rosaceae family, their presence in the *Dryas* genus does not serve as a unique chemodiversity marker. Moreover, the absence of 2-pyrone-4,6-dicarboxylic acid in *D. oxyodonta* extracts is noteworthy, because this compound is a chemotaxonomic marker of the Rosoideae subfamily and has been identified in numerous genera, such as *Agrimonia*, *Filipendula*, *Fragaria*, *Geum*, *Potentilla*, *Rosa*, *Rubus*, and *Sanguisorba* [37,38].

Hydroxycinnamates are common metabolites in the Rosaceae family [39,40,41]. Specifically, caffeic, coumaric, and ferulic acid derivatives are prevalent within the Rosoideae subfamily [42,43,44,45]. Although derivatives of hydroxycinnamic acids were identified in the *D. oxyodonta* extract, there are no records of their discovery in *D. octopetala*, likely owing to the lack of comprehensive knowledge about this species. Procyanidins and catechins, characteristic of the Dryadaceae subfamily, have also been found in other subfamilies, such as Rosoideae and Amygdaloideae, across genera like *Agrimonia* [46], *Prunus* [47], *Malus* [48], and *Pyrus* [49]. Therefore, establishing a distinct chemodiversity pattern for procyanidins and catechins is challenging due to their ubiquitous presence in the plant metabolome. This situation mirrors the one observed with triterpenes. In *D. oxyodonta*, all detected triterpenes belonged to the ursane type, a common triterpenoid found in various members of the Rosaceae family [19,50,51]. Consequently, this triterpenoid type cannot serve as a distinguishing criterion for chemodiversity within the *Dryas* genus.

The presence of derivatives of the flavonols quercetin and kaempferol is a common characteristic of the Rosaceae family, as validated by this study [52]. Additionally, derivatives of the flavonols sexangularetin and corniculatusin are more specific to the *Dryas* genus, identified in *D. oxyodonta* in this study and previously in *D. octopetala* [14]. Previously, the presence of sexangularetin derivatives has been observed in certain representatives of the Amygdaloideae (genera *Crataegus*, *Sorbus*, and *Prunus*) [1,53] and Rosoideae (*Fragaria*) subfamilies [54]. Conversely, corniculatusin derivatives have only been documented within the Rosaceae family in the Dryadoideae subfamily, specifically in the *Cowania*, *Purshia*, and *Dryas* genera [14,55]. Consequently, sexangularetin and corniculatusin glycosides are a phytochemical fingerprint for the *Dryas* genus, because these 8-methoxyflavonol derivatives have not been observed together in other species of the Rosaceae family. A similar occurrence of sexangularetin and corniculatusin in one botanical specimen has previously been reported for *Lotus corniculatus* (Fabaceae) [56,57]. Thus, the phenolic fingerprint profiles, specifically the flavonol glycosides of sexangularetin and corniculatusin, along with the presence of gallotannins and the absence of ellagitannins and 2-pyrone-4,6-dicarboxylic acid, could serve as markers in chemodiversity and potentially chemotaxonomy investigations within the *Dryas* genus. Regarding the question of whether the *Dryas* genus should be included in a separate subfamily, it is important to note that the metabolome of the studied representatives of this genus differs from existing data on the chemical composition patterns of members of other subfamilies within the Rosaceae family. This difference justifies the presence of a separate subfamily for *Dryas*.

### 2.3. Quantitative Analysis of Metabolites in Dryas oxyodonta Extracts

For the quantitative analysis of compounds using HPLC–PDA–ESI–tQ–MS, as well as for a comparative qualitative analysis of the components, samples of *D. oxyodonta* (flowers and leaves) were collected from three different regions of the high-mountain alpine tundra belt in Siberia: Sakha (1000 m), Buryatia (1900 m), and Altai (2300 m) (Table 2). These regions have a sharply continental climate, wide daily and annual temperature fluctuations, and moderate precipitation [58,59].

Procyanidin B1 was identified as the primary compound in both flowers and leaves of *D. oxyodonta* collected from the Sakha region (55.11 and 73.84 mg/g, respectively). Additionally, both flowers and leaves of the same samples exhibited notably high levels of (−)-epicatechin (26.84 and 52.17 mg/g, respectively) and (+)-catechin (14.82 and 45.77 mg/g, respectively). Furthermore, the analysis revealed that the hyperoside content in *D. oxyodonta* flowers exceeded that in leaves by more than fivefold (43.27 vs. 8.21 mg/g, respectively). The primary compound in *D. oxyodonta* flowers from Buryatia was identified as hyperoside (58.33 mg/g), while (−)-epicatechin accumulated in the leaves (73.15 mg/g). High levels of procyanidin B1 and (+)-catechin were also observed in both flowers and leaves, similar to the samples from Sakha. However, the flowers from Altai had a different composition, with the flavonols avicularin (27.82 mg/g) and hyperoside (27.63 mg/g) being the predominant compounds. In the leaves from Altai and those from Buryatia, epicatechin was the dominant compound, with a concentration of 35.11 mg/g. However, similar to the samples from other regions, the flowers and leaves of specimens from Altai also exhibited high levels of procyanidin B1 and (+)-catechin. The overall flavonoid content was highest in the *D. oxyodonta* flowers from Altai samples (123.91 mg/g), and the maximum concentration of phenolic compounds was observed in the flowers from Buryatia (257.80 mg/g).

Notably, all of the studied samples contained potential marker compounds, namely, glycosides of sexangularetin and corniculatusin. Higher concentrations of glycosides were observed in the flowers, while the leaves contained lesser amounts or even trace quantities. This pattern of sexangularetin and corniculatusin derivatives’ accumulation in flowers was previously described for *L. corniculatus*, where the authors proposed it as a taxonomic and ecological characteristic [56]. However, quercetin and kaempferol unsubstituted at the 8 position accumulated maximally in *L. corniculatus* leaves, a trend that was not observed in the leaves of *D. oxyodonta*. Therefore, all populations of *D. oxyodonta* collected in Siberia exhibited a high concentration of phenolic compounds, indicating the presence of antioxidant activity.

### 2.4. Antioxidant Activity of D. oxyodonta Extracts

We conducted a comparative analysis of the antioxidant potential of *D. oxyodonta* extracts derived from flowers and leaves collected from various locations in Siberia (Table 3). The scavenging capacities of the analyzed extracts were assessed using four radical assays: DPPH^•^, ABTS^•+−^, O_2_^•−^, and ^•^OH.

*D. oxyodonta* extracts from flowers collected in Buryatia and Sakha exhibited the most pronounced antioxidant activity, while leaf extracts from the same locations showed less pronounced activity. These results align with expectations because the active extracts are characterized by high levels of potent antioxidants such as catechins, flavonoids, and procyanidins [60,61]. In a previous study, the highest antioxidant activity among the examined species of Arctic plants was found in the *D. octopetala* extract [62]. Consequently, herbal tea made from the vegetative parts of *D. oxyodonta* can be considered to be a valuable source of antioxidants, owing to its high phenolic compound contents.

## 3. Materials and Methods

### 3.1. Plant Material

Plant samples of *Dryas oxyodonta* (flowers and leaves) were collected from three different regions of Siberia: the Republic of Buryatia, Tunkinsky District (21 July 2022; 51°51′47.2″ N 101°43′34.6″ E, 1900 m a.s.l.); the Republic of Sakha (Yakutia), Oymyakonskii District, Tas-Kystabyt Mountains (26 July 2022; 62°59′33.1″ N 145°01′47.8″ E, 1000 m a.s.l.); and the Altai Republic, Ust Koksinkii District (16 July 2022; 49°56′26.7″ N 85°59′11.3″ E, 2300 m a.s.l.). Samples were collected at eight points in the alpine tundra (12–14 samples each). Fresh materials were dried in an IPLS-131 drying oven (Bestek Engineering LLC, Rostov-on-Don, Russia) under the following conditions: convection mode, 40 °C. After the samples reached a humidity of 9–12%, they were stored in an Edry D-450A auto-drying cabinet (Edry Co., Ltd., Taichung, Taiwan) before HPLC separation.

### 3.2. Reagents

The reference standards were purchased from BenchChem (Pasadena, CA, USA): 1-*O*-feruloyl-glucose (Cat. No. B135945); ChemFaces (Wuhan, China): juglanin (Cat. No. CFN96238, ≥98%), trifolin (Cat. No. CFN92079, ≥98%); Scientific Laboratory Supplies (Nottingham, UK): 1-*O*-galloyl glucose (Cat. No. 69288, ≥90%); Selleck Chemicals LLC (Houston, TX, USA): procyanidin B1 (Cat. No. E0240, ≥97%); Sigma-Aldrich (St. Louis, MO, USA): (+)-catechin (Cat. No. 43412, ≥ 99%), corosolic acid (Cat. No. PHL80065, ≥90%), 2,2-diphenyl-1-picrylhydrazyl (Cat. No. D9132), (−)-epicatechin (Cat. No. E4018, ≥98%), lithium perchlorate (Cat. No. 205281, ≥95%), perchloric acid (Cat. No. 244252, ≥70%), procyanidin B2 (Cat. No. 42157, ≥90%), avicularin (Cat. No. 44006, ≥90%), guaijaverin (Cat. No. PHL80986, ≥95%), hyperoside (Cat. No. 83388, ≥97%), reynoutrin (Cat. No. 83390, ≥97%), tormentic acid (Cat. No. PHL85836, ≥95%), Trolox (Cat. No. 648471), ursolic acid (Cat. No. U6753, ≥90%); 1-*O*-caffeoyl-glucose and 1-*O*-*p*-coumaroyl glucose were previously isolated from *Spiraea salicifolia* [63], while 6-*O*-caffeoyl-glucose and 1,6-di-*O*-caffeoyl-glucose were isolated from *Filipendula ulmaria* [64].

### 3.3. Extract Preparation

To prepare the *D. oxyodonta* extracts, 10 g of the ground plant materials (leaves and flowers) was treated twice with 100 mL of 70% methanol using an ultrasonic bath (Sapphire Ltd., Moscow, Russia) with the following sonication parameters: 30 min, 40 °C, frequency 35 kHz, and ultrasound power 100 W. The obtained liquid extracts were combined and centrifuged. The supernatants were filtered through cellulose filters and concentrated until dryness. The yields of the *D. oxyodonta* extracts were 4.3 g (Altai flower extract), 4.6 g (Altai leaf extract), 3.8 (Buryatia flower extract), 4.1 (Buryatia leaf extract), 4.4 g (Sakha flower extract), and 4.2 (Sakha leaf extract). The final dry extracts were conserved at 4 °C for subsequent usage in chromatographic experiments and antioxidant activity studies.

### 3.4. Liquid Chromatography–Mass Spectrometry Detection of Metabolites in D. oxyodonta Extracts

Fingerprinting of *D. oxyodonta* metabolites was carried out using liquid chromatography–mass spectrometry (Table S1). LabSolutions LCMS software (ver. 5.6) was applied to operate the LC-MS system [65]. To identify metabolites, a set of chromatographic and spectral parameters (retention time and UV/MS spectra, respectively) were analyzed in comparison with data from reference compounds, data from the literature, and our own mass spectrometry library. For the preparation of the analyzed solution, *D. oxyodonta* extract (5 mg) was dissolved in methanol in a volumetric flask (5 mL) by shaking, followed by filtration through syringe filters with a pore size of 0.22 μm.

### 3.5. HPLC-PDA-ESI-tQ-MS Quantification of Metabolites in D. oxyodonta Extracts

The quantification of *D. oxyodonta* compounds was performed using liquid chromatography–mass spectrometry conditions (Section 3.4). The full-scan MS peak areas were applied for calculation. Nineteen reference standards were utilized to build calibration curves: 6-*O*-caffeoyl glucose, 1-*O*-galloyl glucose, 1-*O*-caffeoyl glucose, procyanidin B1, (+)-catechin, 1-*O*-*p*-coumaroyl glucose, procyanidin B2, 1-*O*-feruloyl glucose, 1,6-di-*O*-caffeoyl glucose, (−)-epicatechin, hyperoside, guaijaverin, reynoutrin, avicularin, trifolin, juglanin, tormentic acid, corosolic acid, and ursolic acid. The reference compounds were carefully weighed (10 mg), dissolved in 10 mL volumetric flasks with the usage of a methanol–DMSO (1:1) solvent, and then “concentration–mass spectral peak area” graphs were plotted (1–100 µg/mL). The values of the correlation coefficient (r^2^), standard deviation (S_YX_), limit of detection (LOD), limit of quantification (LOQ), and linear range were calculated in Advanced Grapher 2.2 (Alentum Software Inc., Ramat-Gan, Israel), using calibration curve data [66] and the results of three sufficient HPLC runs (Table 4). Intra-day precision, inter-day precision, and the recovery of spiked samples were studied using the known assay [67]. The results were expressed as mean values ± standard deviation (S.D.).

### 3.6. Antioxidant Activity of D. oxyodonta Extracts

Spectrophotometric assays in microplates were used to evaluate the antioxidant potential of *D. oxyodonta* extracts via four radical tests: DPPH^•^ (2,2-diphenyl-1-picrylhydrazyl radical) [68], ABTS^•+–^ (2,2′-azino-bis-(3-ethylbenzothiazoline-6-sulfonic acid) cation radical) [69], O_2_^•–^ (superoxide radical) [70], and ^•^OH (hydroxyl radical) [71]. The results of the DPPH^•^, ABTS^•+–^, O_2_^•–^, and ^•^OH assays were measured as IC_50_ values (the half-maximal inhibitory concentration). To calculate the IC_50_ correlations, “concentration (µg/mL or mg/mL)—antioxidant activity (%)” graphs were used. All tests were carried out five times, and the data obtained were presented as the mean value ± standard deviation (SD).

### 3.7. Statistical Analysis

Statistical analyses were carried out with the usage of one-way analysis of variance. Duncan’s multiple range test was applied to find the significance of the mean differences. Differences were presumed to be statistically significant at *p* < 0.05. The results were provided as the mean ± S.D. Advanced Grapher 2.2 (Alentum Software, Inc., Ramat Gan, Israel) was applied for linear regression analysis, as well as for generating the calibration graphs.

## 4. Conclusions

The *Dryas oxyodonta* species thrives across extensive territories in the subalpine and subarctic zones of the Northern Hemisphere, creating valuable reserves. This study elucidates the chemical composition of this species, which has not been previously examined. In exploring the chemodiversity of *D. oxyodonta*, we established the marker role of certain rare flavonoids. The propensity of *Dryas* to accumulate phenolic compounds, alongside its associated high antioxidant activity, positions the studied species as a promising source of raw medicinal plant materials.

## Figures and Tables

**Table 1:** Retention times (*t*_R_), ultraviolet (UV), and mass spectrometric (ESI-MS) information of **1**–**40** detected in *Dryas oxyodonta*.

No.	*t*_R_, min	UV Pattern ^a^	Molecular Formula	ESI-MS, *m*/*z* (Deprotonated ion [M − H]^−^: Daughter Ions)	Compound [Ref.]	IL ^b^
**1**	1.48	A	C_15_H_18_O_9_	341: 179	6-*O*-Caffeoyl glucose [21]	1
**2**	1.92	B	C_13_H_16_O_10_	331: 169	Galloyl glucose [22]	2
**3**	2.29	B	C_13_H_16_O_10_	331: 169	1-*O*-Galloyl glucose [22]	1
**4**	3.02	A	C_15_H_18_O_9_	341: 179	1-*O*-Caffeoyl glucose [21]	1
**5**	4.96	C	C_30_H_26_O_12_	577	Procyanidin B1 [23]	1
**6**	5.63	C	C_15_H_14_O_6_	289	(+)-Catechin [22]	1
**7**	6.08	D	C_15_H_18_O_8_	325: 163	1-*O*-*p*-Coumaroyl glucose [21]	1
**8**	6.97	C	C_30_H_26_O_12_	577	Procyanidin B2 [23]	1
**9**	7.38	A	C_16_H_20_O_9_	355: 193	1-*O*-Feruloyl glucose [21]	1
**10**	8.08	A	C_24_H_24_O_14_	503: 341, 179	1,6-Di-*O*-caffeoyl glucose [21]	1
**11**	8.31	C	C_15_H_14_O_6_	289	(−)-Epicatechin [22]	1
**12**	9.00	E	C_33_H_40_O_22_	787: 625, 463, 301	Quercetin 3-*O*-trihexoside [24]	2
**13**	9.42	E	C_32_H_38_O_21_	757: 625, 595, 463, 301	Quercetin 3-*O*-pentoside-*O*-dihexoside [25]	2
**14**	10.67	E	C_27_H_30_O_17_	625: 463, 301	Quercetin 3-*O*-dihexoside [24]	2
**15**	11.12	E	C_26_H_28_O_16_	595: 463, 301	Quercetin 3-*O*-pentoside-*O*-hexoside [25]	2
**16**	11.87	F	C_22_H_22_O_13_	493: 331	Corniculatusin *O*-hexoside [26]	2
**17**	12.76	F	C_21_H_20_O_12_	463: 331	Corniculatusin *O*-pentoside [26]	2
**18**	13.0.8	E	C_21_H_20_O_12_	463: 301	Quercetin 3-*O*-galactoside (hyperoside) [27]	1
**19**	13.67	E	C_20_H_18_O_11_	433: 301	Quercetin 3-*O*-arabinopyranoside (guaijaverin) [28]	1
**20**	14.22	G	C_22_H_22_O_12_	477: 315	Sexangularetin *O*-hexoside [29]	2
**21**	14.79	G	C_21_H_20_O_11_	447: 315	Sexangularetin *O*-pentoside [29]	2
**22**	15.55	E	C_20_H_18_O_11_	433: 301	Quercetin 3-*O*-xyloside (reynoutrin) [28]	1
**23**	16.01	E	C_20_H_18_O_11_	433: 301	Quercetin 3-*O*-arabinofuranoside (avicularin) [28]	1
**24**	16.71	H	C_21_H_20_O_11_	447: 285	Kaempferol 3-*O*-galactoside (trifolin) [30]	1
**25**	17.11	H	C_20_H_18_O_10_	417: 285	Kaempferol 3-*O*-arabinofuranoside (juglanin) [30]	1
**26**	17.89	I	C_35_H_34_O_19_	757: 625, 595, 463, 301	Quercetin *O*-caffeoyl-*O*-pentoside-*O*-hexoside [31]	2
**27**	18.09	J	C_31_H_28_O_16_	655: 493, 331	Corniculatusin *O*-caffeoyl-*O*-hexoside [26]	2
**28**	18.19	J	C_30_H_26_O_1_5	625: 463, 331	Corniculatusin *O*-caffeoyl-*O*-pentoside [26]	2
**29**	18.97	I	C_30_H_26_O_15_	625: 463, 301	Quercetin *O*-caffeoyl-*O*-hexoside [31]	2
**30**	19.42	I	C_29_H_24_O_14_	595: 433, 301	Quercetin *O*-caffeoyl-*O*-pentoside [31]	2
**31**	19.63	K	C_31_H_28_O_15_	639: 477, 315	Sexangularetin *O*-caffeoyl-*O*-hexoside [29]	2
**32**	19.94	K	C_30_H_26_O_14_	609: 447, 315	Sexangularetin *O*-caffeoyl-*O*-pentoside [29]	2
**33**	20.02	I	C_29_H_24_O_14_	595: 433, 301	Quercetin *O*-caffeoyl-*O*-pentoside [31]	2
**34**	20.53	I	C_29_H_24_O_14_	595: 433, 301	Quercetin *O*-caffeoyl-*O*-pentoside [31]	2
**35**	20.82	L	C_30_H_48_O_5_	487	Tormentic acid [19]	1
**36**	21.02	L	C_30_H_48_O_4_	471	Corosolic acid [19]	1
**37**	21.12	L	C_30_H_48_O_3_	455	Ursolic acid [19]	1
**38**	21.20	M	C_30_H_26_O_14_	609: 447, 285	Kaempferol *O*-caffeoyl-*O*-hexoside [30]	2
**39**	21.53	L	C_30_H_48_O_3_	455	Ursolic acid isomer [19]	2
**40**	21.89	M	C_29_H_24_O_13_	579: 417, 285	Kaempferol *O*-caffeoyl-*O*-pentoside [30]	2

^a^ UV patterns: A―328 ± 2 nm; B―217 ± 2, 277 ± 2 nm; C―278 ± 1 nm; D―314 ± 1 nm; E―254 ± 1, 265 ± 1, 352 ± 2 nm; F―273 ± 2, 360 ± 2 nm; G―270 ± 2, 356 ± 2 nm; H―250 ± 3, 350 ± 2 nm; I―253 ± 1, 265 ± 1, 333 ± 3 nm; J―273 ± 2, 343 ± 3 nm; K―271 ± 2, 335 ± 2 nm; L―205 ± 4 nm; M―252 ± 3, 332 ± 2 nm. ^b^ Identification levels: 1 identified compounds after comparison of UV and MS data and retention times with reference standards; 2 putatively annotated compounds after comparison of UV and MS data with data from the literature.

**Table 2:** Contents of compounds **1**–**40** in leaves and flowers of *Dryas oxyodonta* collected from three locations in Siberia, mg/100 g FW (±S.D.).

No.	Compound	Sakha 1000 m (*n* = 14)		Buryatia 1900 m (*n* = 14)		Altai 2300 m (*n* = 12)	
		Flowers	Leaves	Flowers	Leaves	Flowers	Leaves
1	6-*O*-Caffeoyl glucose	0.93 ± 0.02	0.90 ± 0.02	1.03 ± 0.02	tr.	tr.	tr.
2	Galloyl glucose	1.87 ± 0.03	5.11 ± 0.11	1.69 ± 0.04	2.83 ± 0.05	tr.	tr.
3	1-*O*-Galloyl glucose	2.14 ± 0.04	3.73 ± 0.07	1.53 ± 0.03	1.65 ± 0.03	0.93 ± 0.02	tr.
4	1-*O*-Caffeoyl glucose	3.75 ± 0.06	0.93 ± 0.02	4.83 ± 0.10	1.27 ± 0.02	1.11 ± 0.02	1.24 ± 0.02
5	Procyanidin B1	55.11 ± 0.99	73.84 ± 1.40	42.14 ± 0.88	40.83 ± 0.86	21.15 ± 0.38	20.53 ± 0.41
6	(+)-Catechin	14.82 ± 0.27	45.77 ± 0.96	26.84 ± 0.51	39.14 ± 0.82	14.22 ± 0.27	22.83 ± 0.43
7	1-*O*-*p*-Coumaroyl glucose	2.63 ± 0.04	1.73 ± 0.04	3.79 ± 0.08	0.61 ± 0.01	0.27 ± 0.01	tr.
8	Procyanidin B2	19.25 ± 0.35	29.16 ± 0.61	10.06 ± 0.19	11.29 ± 0.24	9.57 ± 0.19	8.27 ± 0.17
9	1-*O*-Feruloyl glucose	0.83 ± 0.02	tr.	0.93 ± 0.02	tr.	0.52 ± 0.01	tr.
10	1,6-Di-*O*-caffeoyl glucose	0.24 ± 0.00	tr.	0.12 ± 0.00	tr.	0.14 ± 0.00	tr.
11	(−)-Epicatechin	26.84 ± 0.51	52.17 ± 1.04	42.03 ± 0.84	73.15 ± 1.61	22.82 ± 0.46	35.11 ± 0.70
12	Quercetin 3-*O*-trihexoside	1.01 ± 0.02	tr.	0.12 ± 0.00	tr.	1.09 ± 0.02	0.14 ± 0.00
13	Quercetin 3-*O*-pentoside-*O*-dihexoside	0.67 ± 0.01	tr.	0.09 ± 0.00	tr.	1.72 ± 0.04	0.21 ± 0.00
14	Quercetin 3-*O*-dihexoside	1.43 ± 0.03	tr.	0.53 ± 0.01	tr.	4.35 ± 0.09	0.41 ± 0.01
15	Quercetin 3-*O*-pentoside-*O*-hexoside	1.24 ± 0.02	tr.	0.72 ± 0.02	tr.	2.09 ± 0.04	0.52 ± 0.01
16	Corniculatusin 3-*O*-galactoside	7.83 ± 0.15	2.73 ± 0.05	5.79 ± 0.12	0.50 ± 0.01	0.11 ± 0.00	0.08 ± 0.00
17	Corniculatusin 3-*O*-arabinoside	6.33 ± 0.11	1.29 ± 0.03	4.25 ± 0.09	0.41 ± 0.01	0.23 ± 0.00	0.09 ± 0.00
18	Quercetin 3-*O*-galactoside (hyperoside)	43.27 ± 0.69	8.21 ± 0.16	58.33 ± 1.22	12.63 ± 0.21	27.63 ± 0.58	2.63 ± 0.06
19	Quercetin 3-*O*-arabinopyranoside (guaijaverin)	10.52 ± 0.19	5.27 ± 0.10	14.63 ± 0.28	6.24 ± 0.12	22.85 ± 0.48	11.81 ± 0.26
20	Sexangularetin 3-*O*-galactoside	1.52 ± 0.03	0.40 ± 0.01	0.84 ± 0.01	tr.	0.28 ± 0.01	0.15 ± 0.00
21	Sexangularetin 3-*O*-arabinoside	2.16 ± 0.04	0.52 ± 0.01	0.92 ± 0.02	tr.	0.37 ± 0.01	0.06 ± 0.00
22	Quercetin 3-*O*-xyloside (reynoutrin)	5.27 ± 0.10	tr.	6.83 ± 0.14	0.83 ± 0.01	11.64 ± 0.23	9.83 ± 0.17
23	Quercetin 3-*O*-arabinofuranoside (avicularin)	9.83 ± 0.17	1.53 ± 0.03	14.95 ± 0.31	3.86 ± 0.08	27.82 ± 0.53	5.22 ± 0.09
24	Kaempferol 3-*O*-galactoside (trifolin)	4.03 ± 0.08	0.93 ± 0.02	5.29 ± 0.09	1.95 ± 0.04	9.65 ± 0.18	2.10 ± 0.04
25	Kaempferol 3-*O*-arabinofuranoside (juglanin)	0.52 ± 0.01	tr.	1.89 ± 0.04	0.52 ± 0.01	3.76 ± 0.07	1.52 ± 0.03
26	Quercetin *O*-caffeoyl-*O*-pentoside-*O*-hexoside	0.41 ± 0.01	tr.	1.22 ± 0.02	0.21 ± 0.00	1.73 ± 0.03	tr.
27	Corniculatusin *O*-caffeoyl-*O*-hexoside	2.67 ± 0.05	0.63 ± 0.01	0.72 ± 0.02	tr.	tr.	0.27 ± 0.00
28	Corniculatusin *O*-caffeoyl-*O*-pentoside	1.54 ± 0.03	1.14 ± 0.03	0.70 ± 0.01	tr.	0.44 ± 0.01	0.19 ± 0.00
29	Quercetin *O*-caffeoyl-*O*-hexoside	0.24 ± 0.00	tr.	0.63 ± 0.01	0.08 ± 0.00	1.14 ± 0.02	tr.
30	Quercetin *O*-caffeoyl-*O*-pentoside	0.12 ± 0.00	tr.	0.69 ± 0.01	0.05 ± 0.00	1.52 ± 0.03	tr.
31	Sexangularetin *O*-caffeoyl-*O*-hexoside	0.53 ± 0.01	0.04 ± 0.00	0.08 ± 0.00	tr.	0.02 ± 0.00	tr.
32	Sexangularetin *O*-caffeoyl-*O*-pentoside	2.67 ± 0.06	0.22 ± 0.00	1.10 ± 0.02	0.09 ± 0.00	tr.	tr.
33	Quercetin *O*-caffeoyl-*O*-pentoside	tr.	tr.	0.54 ± 0.01	tr.	0.97 ± 0.02	tr.
34	Quercetin *O*-caffeoyl-*O*-pentoside	tr.	tr.	0.63 ± 0.01	tr.	1.49 ± 0.03	tr.
35	Tormentic acid	6.85 ± 0.14	7.39 ± 0.14	2.94 ± 0.06	3.85 ± 0.08	1.41 ± 0.03	2.53 ± 0.05
36	Corosolic acid	2.74 ± 0.06	3.67 ± 0.07	0.53 ± 0.01	1.02 ± 0.02	0.92 ± 0.02	1.14 ± 0.02
37	Ursolic acid	2.22 ± 0.04	1.29 ± 0.03	0.60 ± 0.01	0.42 ± 0.01	0.57 ± 0.01	1.69 ± 0.03
38	Kaempferol *O*-caffeoyl-*O*-hexoside	tr.	tr.	0.63 ± 0.01	tr.	0.90 ± 0.02	0.52 ± 0.01
39	Ursolic acid isomer	1.14 ± 0.02	0.93 ± 0.02	0.83 ± 0.02	0.92 ± 0.02	1.14 ± 0.02	0.95 ± 0.02
40	Kaempferol *O*-caffeoyl-*O*-pentoside	tr.	tr.	0.69 ± 0.01	tr.	2.11 ± 0.04	0.92 ± 0.02
	Total cinnamoyl glucoses	8.38	3.56	10.70	1.88	2.04	1.24
	Total galloyl glucoses	4.01	8.84	3.22	4.48	0.93	tr.
	Total procyanidins	74.36	103.00	52.20	52.12	30.72	28.80
	Total catechins	41.66	97.94	68.87	112.29	37.04	57.94
	Total flavonoids, including:	103.81	22.91	122.81	27.37	123.91	36.67
	Kaempferol glucosides	4.55	0.93	8.50	2.47	16.42	5.06
	Quercetin glucosides	74.01	15.01	99.91	23.90	106.04	30.77
	Sexangularetin glucosides	6.88	1.18	2.94	0.09	0.67	0.21
	Corniculatusin glucosides	18.37	5.79	11.46	0.91	0.78	0.63
	Total phenolics	232.22	236.25	257.80	198.14	194.64	124.65
	Total triterpenes	12.95	13.28	4.90	6.21	4.04	6.31

tr.—traces (<limit of quantification).

**Table 3:** Radical scavenging activity of *D. oxyodonta* extracts.

Extracts	DPPH^• a^	ABTS^•+ a^	O_2_^•– a^	^•^OH ^a^
SFE	9.23 ± 0.18	5.04 ± 0.10	26.13 ± 0.52	14.92 ± 0.29
SLE	11.08 ± 0.22	7.12 ± 0.14	37.16 ± 0.74	8.08 ± 0.16
BFE	8.67 ± 0.17	3.99 ± 0.08	21.59 ± 0.43	12.78 ± 0.25
BLE	11.90 ± 0.23	4.36 ± 0.09	41.14 ± 0.82	19.36 ± 0.39
AFE	15.01 ± 0.30	8.07 ± 0.16	58.22 ± 1.16	36.05 ± 0.72
ALE	18.33 ± 0.36	10.85 ± 0.21	73.01 ± 1.46	49.32 ± 0.99
Trolox ^b^	10.17 ± 0.20	4.67 ± 0.09	109.28 ± 2.18	16.37 ± 0.33

SFE—Sakha flower extract. SLE—Sakha leaf extract. BFE—Buryatia flower extract. BLE—Buryatia leaf extract. AFE—Altai flower extract. ALE—Altai leaf extract. ^a^ IC_50_, µg/mL ± SD. ^b^ Reference compound.

**Table 4:** Regression equations, correlation coefficients (r^2^), standard deviation (S_yx_), limits of detection (LOD), limits of quantification (LOQ), and linear ranges for 19 reference standards used for HPLC-MS quantification.

Compound	Regression Equation ^a^		r^2^	S_yx_	LOD/LOQ (µg/mL)	Linear Range (µg/mL)	RSD% (Intra-Day)	RSD% (Inter-Day)	Recovery of Spiked Sample REC%
	*a*	*b* × 10^6^							
6-*O*-Caffeoyl glucose	1.3387	−0.0284	0.9981	9.50 × 10^−2^	0.23/0.71	1.0–100.0	1.45	1.92	101.27
1-*O*-Galloyl glucose	1.3586	−0.0663	0.9987	9.69 × 10^−2^	0.24/0.71	1.0–100.0	0.97	1.16	98.34
1-*O*-Caffeoyl glucose	1.5824	−0.1078	0.9965	16.25 × 10^−2^	0.34/1.03	1.0–100.0	0.89	1.29	100.64
Procyanidin B1	1.3722	−0.0829	0.9973	9.93 × 10^−2^	0.24/0.72	1.0–100.0	1.09	1.36	99.12
(+)-Catechin	0.9562	−0.0521	0.9971	7.79 × 10^−2^	0.27/0.82	1.0–100.0	1.23	1.54	100.07
1-*O*-*p*-Coumaroyl glucose	1.4238	−0.0891	0.9901	7.33 × 10^−2^	0.17/0.52	1.0–100.0	1.38	1.88	101.14
Procyanidin B2	1.3620	−0.0820	0.9961	9.91 × 10^−2^	0.21/0.72	1.0–100.0	1.47	1.64	100.78
1-*O*-Feruloyl glucose	1.5152	−0.0523	0.9979	12.67 × 10^−2^	0.28/0.84	1.0–100.0	1.33	1.61	100.11
1,6-Di-*O*-caffeoyl glucose	1.7552	−0.0569	0.9982	8.89 × 10^−2^	0.18/0.51	1.0–100.0	1.40	1.98	100.39
(−)-Epicatechin	1.0828	−0.0456	0.9973	6.85 × 10^−2^	0.21/0.63	1.0–100.0	1.12	1.42	99.23
Hyperoside	1.4689	−0.3641	0.9990	5.69 × 10^−2^	0.12/0.38	1.0–100.0	1.22	1.43	101.22
Guaijaverin	1.3436	−0.4406	0.9981	17.58 × 10^−2^	0.43/1.31	1.0–100.0	0.99	1.60	102.55
Reynoutrin	1.5364	−0.3614	0.9927	10.07 × 10^−2^	0.22/0.66	1.0–100.0	1.34	1.78	102.03
Avicularin	1.4041	−0.3270	0.9992	14.02 × 10^−2^	0.33/1.00	1.0–100.0	1.31	1.57	99.83
Trifolin	2.0859	−0.9171	0.9980	6.18 × 10^−2^	0.03/0.09	1.0–100.0	1.23	1.83	101.23
Juglanin	2.2126	−0.5160	0.9987	8.11 × 10^−2^	0.12/0.37	1.0–100.0	1.08	1.60	98.33
Tormentic acid	1.5330	−0.0863	0.9985	4.15 × 10^−2^	0.09/0.27	1.0–100.0	1.39	1.78	100.09
Corosolic acid	2.3312	−0.4563	0.9803	14.92 × 10^−2^	0.21/0.64	1.0–100.0	1.24	1.85	101.40
Ursolic acid	1.2820	−0.9634	0.9697	11.64 × 10^−2^	0.30/0.91	1.0–100.0	1.41	1.91	99.54

^a^ Regression equation: *y = a* × *x + b.*

## Data Availability

Data are contained within the article.

## Supplementary Materials

The following supporting information are available at: https://www.mdpi.com/article/10.3390/plants13060868/s1. Figure S1: Structures of compounds identified in *Dryas oxyodonta*. Table S1: Conditions for liquid chromatography–mass spectrometry detection of metabolites in *Dryas oxyodonta* extracts.

## References

[1] Challice J.S. (1974). Rosaceae chemotaxonomy and the origins of the Pomoideae. Bot. J. Linn. Soc..

[2] Takhtajan A. (1997). Diversity and Classification of Flowering Plants.

[3] Shulaev V., Korban S.S., Sosinski B., Abbott A.G., Aldwinckle H.S., Folta K.M., Iezzoni A., Main D., Arús P., Dandekar A.M. (2008). Multiple models for Rosaceae genomics. Plant Physiol..

[4] Okuda T., Yoshida T., Hatano T., Iwasaki M., Kubo M., Orime T., Yoshizaki M., Naruhashi N. (1992). Hydrolysable tannins as chemotaxonomic markers in the Rosaceae. Phytochemistry.

[5] Potter D., Eriksson T., Evans R.C., Oh S., Smedmark J.E.E., Morgan D.R., Kerr M., Robertson K.R., Arsenault M., Dickinson T.A. (2007). Phylogeny and classification of Rosaceae. Plant Syst. Evol..

[6] Watson L., Dallwitz M.J. (1994). The families of flowering plants. Interactive identification and information retrieval on CD-ROM version 1.0 1993, and colour illustrated manual. Nord. J. Bot..

[7] Sedelnikov V.P. (2015). High-mountain vegetation of North Asia: Dryad tundras. Contemp. Probl. Ecol..

[8] McGraw J.B., Antanovics J. (1983). Experimental ecology of *Dryas octopetala* ecotypes: I. Ecotypic differentiation and life cycle stages of selection. J. Ecol..

[9] Komarov V.L. (1941). Flora of USSR.

[10] Plants of the World Online. https://powo.science.kew.org/taxon/urn:lsid:ipni.org:names:724676-1.

[11] Makarov A.A. (1974). Plant Medical Remedies of Yakut Traditional Medicine.

[12] Batorova S.M., Yakovlev G.P., Aseeva T.A. (2013). Reference-Book of Traditional Tibetan Medicine Herbs.

[13] Pangon J.F., Jay M., Voirin B. (1974). Les flavonoides du *Dryas octopetala*. Phytochemistry.

[14] Servettaz O., Colombo M.L., De Bernardi M., Uberti E., Vidari G., Vita-Finzi P. (1984). Flavonol glycosides from *Dryas octopetala*. J. Nat. Prod..

[15] Petelka J., Plagg B., Säumel I., Zerbe S. (2020). Traditional medicinal plants in South Tyrol (northern Italy, southern Alps): Biodiversity and use. J. Ethnobiol. Ethnomed..

[16] Manninen M., Karonen M., Salminen J.-P. (2022). Chemotaxonomic markers for the leaf buds of common Finnish trees and shrubs: A rapid UHPLC MS fingerprinting tool for species identification. Molecules.

[17] Kashchenko N.I., Olennikov D.N. (2020). Phenolome of Asian agrimony tea (*Agrimonia asiatica* Juz., Rosaceae): LC-MS profile, α-glucosidase inhibitory potential and stability. Foods.

[18] Kashchenko N.I., Olennikov D.N., Chirikova N.K. (2021). Metabolites of Siberian raspberries: LC-MS profile, seasonal variation, antioxidant activity and, thermal stability of *Rubus matsumuranus* phenolome. Plants.

[19] Kashchenko N.I., Olennikov D.N., Chirikova N.K. (2023). Metabolites of *Geum aleppicum* and *Sibbaldianthe bifurca*: Diversity and α-glucosidase inhibitory potential. Metabolites.

[20] Sumner L.W., Amberg A., Barrett D., Beale M.H., Beger R., Daykin C.A., Fan T.W.M., Fiehn O., Goodacre R., Griffin J.L. (2007). Proposed minimum reporting standards for chemical analysis: Chemical Analysis Working Group (CAWG) Metabolomics Standards Initiative (MSI). Metabolomics.

[21] Vallverdú-Queralt A., Jáuregui O., Medina-Remón A., Andrés-Lacueva C., Lamuela-Raventós R.M. (2010). Improved characterization of tomato polyphenols using liquid chromatography/electrospray ionization linear ion trap quadrupole Orbitrap mass spectrometry and liquid chromatography/electrospray ionization tandem mass spectrometry. Rapid Commun. Mass Spectrom..

[22] Olennikov D.N., Chirikova N.K., Vasilieva A.G., Fedorov I.A. (2020). LC-MS profile, gastrointestinal and gut microbiota stability and antioxidant activity of *Rhodiola rosea* herb metabolites: A comparative study with subterranean organs. Antioxidants.

[23] Rockenbach I.I., Jungfer E., Ritter C., Santiago-Schübel B., Thiele B., Fett R., Galensa R. (2012). Characterization of flavan-3-ols in seeds of grape pomace by CE, HPLC-DAD-MSn and LC-ESI-FTICR-MS. Food Res. Int..

[24] Qu C., Fu F., Lu K., Zhang K., Wang R., Xu X., Wang M., Lu J., Wan H., Zhanglin T. (2013). Differential accumulation of phenolic compounds and expression of related genes in black- and yellow-seeded *Brassica napus*. J. Exp. Bot..

[25] Boso S., Gago P., Santiago J.-L., Álvarez-Acero I., Martinez Bartolomé M.-A., Martínez M.-C. (2022). Polyphenols in the waste water produced during the hydrodistillation of ‘Narcea roses’ cultivated in the Cibea river valley (Northern Spain). Horticulturae.

[26] Iwashina T., Katoh N. (2018). Qualitative and quantitative variation of anthocyanins and flavonols among the different organs of *Cercidiphyllum japonicum*. Bull. Natl. Mus. Nat. Sci..

[27] Yuan W., Wang J., An X., Dai M., Jiang Z., Zhang L., Yu S., Huang X. (2021). UPLC-MS/MS method for the determination of hyperoside and application to pharmacokinetics study in rat after different administration routes. Chromatographia.

[28] Fernandes P.A.R., Ferreira S.S., Bastos R., Ferreira I., Cruz M.T., Pinto A., Coelho E., Passos C.P., Coimbra M.A., Cardoso S.M. (2019). Apple pomace extract as a sustainable food ingredient. Antioxidants.

[29] Zymone K., Raudone L., Žvikas V., Jakštas V., Janulis V. (2022). Phytoprofiling of *Sorbus* L. inflorescences: A valuable and promising resource for phenolics. Plants.

[30] Owczarek A., Kołodziejczyk-Czepas J., Marczuk P., Siwek J., Wąsowicz K., Olszewska M.A. (2021). Bioactivity potential of *Aesculus hippocastanum* L. flower: Phytochemical profile, antiradical capacity and protective effects on human plasma components under oxidative/nitrative stress in vitro. Pharmaceuticals.

[31] López-Angulo G., Montes-Avila J., Díaz-Camacho S.P., Vega-Aviña R., López-Valenzuela J.Á., Delgado-Vargas F. (2018). Comparison of terpene and phenolic profiles of three wild species of *Echeveria* (Crassulaceae). J. Appl. Bot. Food Qual..

[32] Clifford M.N., Scalbert A. (2000). Ellagitannins–Nature, occurrence and dietary burden. J. Sci. Food Agric..

[33] Camann J., Denzel K., Schilling G., Gross G.G. (1989). Biosynthesis of gallotannins: β-Glucogallin-dependent formation of 1,2,3,4,6-pentagalloylglucose by enzymatic galloylation of 1,2,3,6-tetragalloylglucose. Arch. Biochem. Biophys..

[34] Ishimaru K., Hirose M., Takahashi K., Koyama K., Shimomura K. (1990). Tannin production in root culture of *Sanguisorba officinalis*. Phytochemistry.

[35] Schulenburg K., Feller A., Hoffmann T., Schecker J.H., Martens S., Schwab W. (2016). Formation of β-glucogallin, the precursor of ellagic acid in strawberry and raspberry. J. Exp. Bot..

[36] Eriksson T., Hibbs M.S., Yoder A.D., Delwiche C.F., Donoghue M.J. (2003). The phylogeny of Rosoideae (Rosaceae) based on sequences of the internal transcribed spacers (its) of nuclear ribosomal DNA and the *TRNL/F* region of chloroplast DNA. Int. J. Plant Sci..

[37] Wilkes S., Glasl H. (2001). Isolation, characterization, and systematic significance of 2-pyrone-4,6-dicarboxylic acid in Rosaceae. Phytochemistry.

[38] Morgan D.R., Soltis D.E., Robertson K.R. (1994). Systematic and evolutionary implications of *rbc*L sequence variation in Rosaceae. Am. J. Bot..

[39] Hameed A., Liu Z., Wu H., Zhong B., Ciborowski M., Suleria H.A.R. (2022). A comparative and comprehensive characterization of polyphenols of selected fruits from the Rosaceae family. Metabolites.

[40] Neelam, Khatkar A., Sharma K.K. (2020). Phenylpropanoids and its derivatives: Biological activities and its role in food, pharmaceutical and cosmetic industries. Crit. Rev. Food Sci. Nutr..

[41] Vasco C., Riihinen K., Ruales J., Kamal-Eldin A. (2009). Phenolic compounds in Rosaceae fruits from Ecuador. J. Agric. Food Chem..

[42] Patras M.A., Jaiswal R., McDougall G.J., Kuhnert N. (2018). Profiling and quantification of regioisomeric caffeoyl glucoses in berry fruits. J. Agric. Food Chem..

[43] Hussein S.A.M., Ayoub N.A., Nawwar M.A.M. (2003). Caffeoyl sugar esters and an ellagitannin from *Rubus sanctus*. Phytochemistry.

[44] Fecka I., Bednarska K., Włodarczyk M. (2022). *Fragaria* × *ananassa* cv. Senga Sengana leaf: An agricultural waste with antiglycation potential and high content of ellagitannins, flavonols, and 2-pyrone-4,6-dicarboxylic acid. Molecules.

[45] Jiang L., Lu M., Rao T., Liu Z., Wu X., An H. (2022). Comparative analysis of fruit metabolome using widely targeted metabolomics reveals nutritional characteristics of different *Rosa roxburghii* genotypes. Foods.

[46] Granica S., Kluge H., Horn G., Matkowski A., Kiss A.K. (2015). The phytochemical investigation of *Agrimonia eupatoria* L. and *Agrimonia procera* Wallr. as valid sources of *Agrimoniae* herba—The pharmacopoeial plant material. J. Pharm. Biomed. Anal..

[47] Ortega-Vidal J., Ruiz-Martos L., Salido S., Altarejos J. (2023). Proanthocyanidins in pruning wood extracts of four European plum (*Prunus domestica* L.) cultivars and their hLDHA inhibitory activity. Chem. Biodivers..

[48] Sunagawa T., Shimizu T., Kanda T., Tagashira M., Sami M., Shirasawa T. (2011). Procyanidins from apples (*Malus pumila* Mill.) extend the lifespan of *Caenorhabditis elegans*. Planta Med..

[49] Jeong D.E., Cho J.Y., Lee Y.G., Jeong H.Y., Lee H.J., Moon J.H. (2017). Isolation of five proanthocyanidins from pear (*Pyrus pyrifolia* Nakai) fruit peels. Food Sci. Biotechnol..

[50] Uto T., Sakamoto A., Tung N.H., Fujiki T., Kishihara K., Oiso S., Kariyazono H., Morinaga O., Shoyama Y. (2013). Anti-proliferative activities and apoptosis induction by triterpenes derived from *Eriobotrya japonica* in human leukemia cell lines. Int. J. Mol. Sci..

[51] Quan X.X., Huang Y.Y., Chen L., Yuan J.Q. (2022). Traditional uses, phytochemical, pharmacology, quality control and modern applications of two important Chinese medicines from *Rosa laevigata* Michx.: A review. Front. Pharmacol..

[52] Bate-Smith E.C. (1965). Investigation of the chemistry and taxonomy of sub-tribe Quillajeae of the Rosaceae using comparisons of fresh and herbarium material. Phytochemistry.

[53] Sołtys A., Galanty A., Podolak I. (2020). Ethnopharmacologically important but underestimated genus *Sorbus*: A comprehensive review. Phytochem. Rev..

[54] Baek Y.S., Song N.Y., Nam T.G., Kim D.-O., Kang H.-C., Kwon O.-K., Baek N.-I. (2015). Flavonoids from *Fragaria ananassa* calyx and their antioxidant capacities. J. Korean Soc. Appl. Biol. Chem..

[55] Koehler D.L., Smith D.M. (1981). Hybridization between *Cowania mexicana* var. *stansburiana* and *Purshia glandulosa* (Rosaceae). Madroño.

[56] Jay M., De Luca V., Ibrahim R. (1983). Meta-methylation of flavonol rings A (8-) and B (3’-) is catalysed by two distinct *O*-methyltransferases in *Lotus corniculatus*. Z. Naturforsch. C.

[57] García-Calderón M., Pérez-Delgado C.M., Palove-Balang P., Betti M., Márquez A.J. (2020). Flavonoids and isoflavonoids biosynthesis in the model legume *Lotus japonicus*; connections to nitrogen metabolism and photorespiration. Plants.

[58] Kharlamova N., Sukhova M., Chlachula J. (2019). Present climate development in Southern Siberia: A 55-year weather observations record. IOP Conf. Ser. Earth Environ. Sci..

[59] Watanabe T., Matsuyama H., Kuzhevskaia I., Nechepurenko O., Chursin V., Zemtsov V. (2023). Long-Term trends of extreme climate indexes in the Southern part of Siberia in comparison with those of surrounding regions. Atmosphere.

[60] Patanè G.T., Putaggio S., Tellone E., Barreca D., Ficarra S., Maffei C., Calderaro A., Laganà G. (2023). Catechins and proanthocyanidins involvement in metabolic syndrome. Int. J. Mol. Sci..

[61] Speisky H., Shahidi F., Costa de Camargo A., Fuentes J. (2022). Revisiting the oxidation of flavonoids: Loss, conservation or enhancement of their antioxidant properties. Antioxidants.

[62] Singh P., Singh S.M., D’Souza L.M., Wahidullah S. (2012). Phytochemical profiles and antioxidant potential of four Arctic vascular plants from Svalbard. Polar Biol..

[63] Olennikov D.N., Kashchenko N.I. (2017). Spireasalicin, a new acylated quercetin glycoside from *Spiraea salicifolia*. Chem. Nat. Comp..

[64] Olennikov D.N., Kruglova M.Y. (2013). A new quercetin glycoside and other phenolic compounds from the genus *Filipendula*. Chem. Nat. Comp..

[65] LabSolutions. https://www.shimadzu.eu/labsolutions-0.

[66] Olennikov D.N., Chirikova N.K., Kashchenko N.I., Nikolaev V.M., Kim S.-W., Vennos C. (2018). Bioactive phenolics of the genus *Artemisia* (Asteraceae): HPLC-DAD-ESI-TQ-MS/MS profile of the Siberian species and their inhibitory potential against α-amylase and α-glucosidase. Front. Pharmacol..

[67] Olennikov D.N., Zilfikarov I.N., Penzina T.A. (2013). Use of microcolumn HPLC for analysis of aloenin in *Aloe arborescens* raw material and related drugs. Pharm. Chem. J..

[68] Asker M.M.S., Shawky B.T. (2010). Structural characterization and antioxidant activity of an extracellular polysaccharide isolated from *Brevibacterium otitidis* BTS 44. Food Chem..

[69] Ding H., Chou T., Liang C. (2010). Antioxidant and antimelanogenic properties of rosmarinic acid methyl ester from *Origanum vulgare*. Food Chem..

[70] Yao Y., Chen S., Li H. (2021). An improved system to evaluate superoxide-scavenging effects of bioflavonoids. ChemistryOpen.

[71] Luqman S., Kumar R. (2012). Importance of deoxyribose degradation assay for evaluating hydroxyl radical scavenging activity of *Punica* extract. Int. J. Food Prop..

