# Supplementary material for: Chemodiversity of Arctic Plant Dryas oxyodonta: LC-MS Profile and Antioxidant Activity
Source: Plants (Basel). 2024 Mar 18;13(6):868. doi: 10.3390/plants13060868 (PMC10975042; doi:10.3390/plants13060868)
Supplement: Supplementary file 1 [file plants-13-00868-s001.zip › plants-2917534-supplementary.pdf]

Supplementary Material

Chemodiversity of Arctic Plant *Dryas oxyodonta*: LC-MS Profile and Antioxidant Activity

Nina I. Kashchenko [1,*], Daniil N. Olennikov [1] and Nadezhda K. Chirikova [2]

[1] Laboratory of Biomedical Research, Institute of General and Experimental Biology, Siberian Division, Russian Academy of Science, 6 Sakh'yanovoy Street, 670047 Ulan-Ude, Russia; olennikovdn@mail.ru
[2] Department of Biochemistry and Biotechnology, North-Eastern Federal University, 58 Belinsky Street, 677027 Yakutsk, Russia; hofnung@mail.ru
* Correspondence: ninkk@mail.ru; Tel.: +7-983-421-73-40

Content:

Figure S1. Structures of compounds identified in *Dryas oxyodonta*.

Table S1. Conditions for liquid chromatography mass spectrometry detection of metabolites in *Dryas oxyodonta* extracts

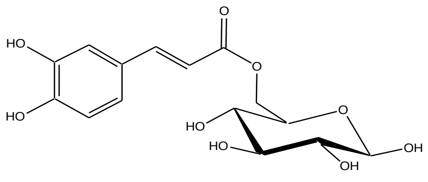

6-*O*-Caffeoyl glucose

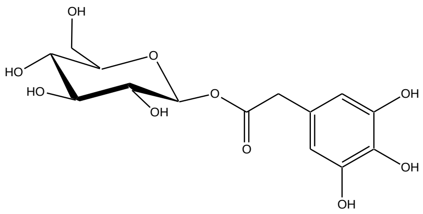

1-*O*-Galloyl glucose

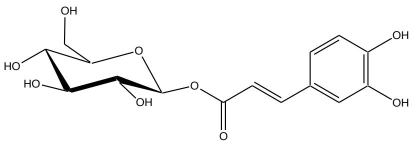

1-*O*-Caffeoyl glucose

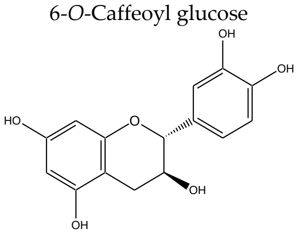

(+)-Catechin

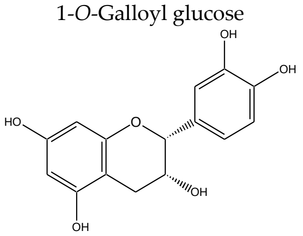

(-)-Epicatechin

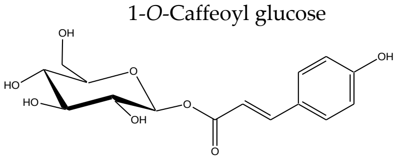

1-*p*-Coumaroyl glucose

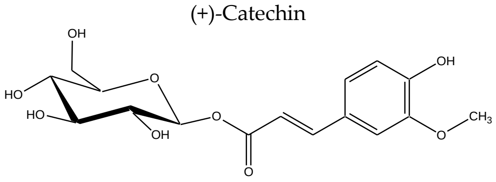

1-*O*-Feruloyl glucose

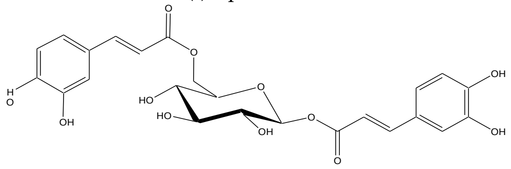

1,6-Di-*O*-Caffeoyl glucose

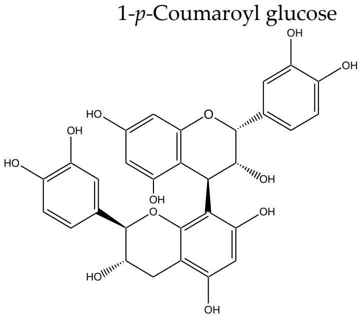

Procyanidin B1

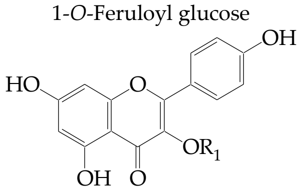

Kaempferol 3-*O*-arabinoside: R_1 = Ara; R_2 = H
Kaempferol 3-*O*-galactoside: R_1 = Gal; R_2 = H

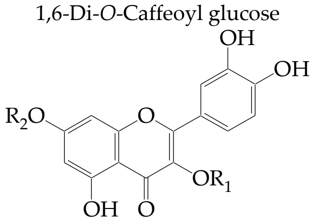

Quercetin 3-*O*-galactoside: R_1 = Gal; R_2 = H
Quercetin 3-*O*-arabinoside: R_1 = Ara; R_2 = H
Quercetin 3-*O*-xyloside: R_1 = Xyl; R_2 = H

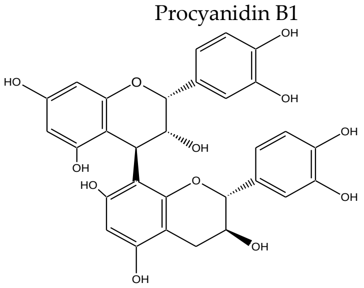

Procyanidin B2

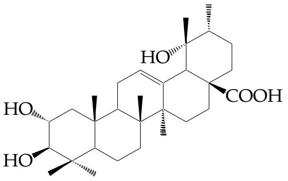

Tormentic acid

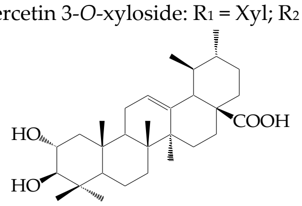

Corosolic acid

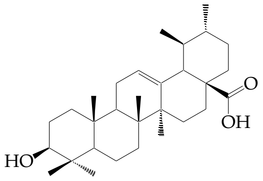

Ursolic acid

Figure S1. Structures of compounds identified in *Dryas oxyodonta*. Abbreviation used: Gal – galactose; Ara – arabinose; Xyl – xylose.

Table S1. Conditions for liquid chromatography mass spectrometry detection of metabolites in *Dryas oxyodonta* extracts

Liquid chromatograph	LC-20 Prominence liquid chromatograph (Shimadzu, Columbia, MD, USA)
Photodiode array detector	SPD-M30A
Mass spectrometer	LCMS 8050 triple quadrupole
Column	GLC Mastro column (2.1 × 150 mm, 3 μm; Shimadzu, Kyoto, Japan)
Eluents	0.25% formic acid in water (A) and 0.25% formic acid in acetonitrile (B)
Gradient elution program	0–3 min (5–9% B), 3–6 min (9–13% B), 6–10 min (13–35% B), 10–15 min (35–58% B), 15–18 min (58–79% B), 18–20 min (79–95% B), 20–25 min (95–5% B)
Injection volume	1 μL
Flow rate	100 μL/min
Column temperature	28 °C
Spectral range of photodiode array detection	200–600 nm
Mass spectrometric mode detection	Negative electrospray ionization
Source voltage	3 kV
Collision energy	−35 eV
Scanning range	100–2000 *m/z*
ESI interface temperature	300 °C
Desolvation line temperature	250 °C
Heat block temperature	400 °C
Flow rate of nebulazing gas N_2	3 L/min
Flow rate of heating gas	10 L/min
Flow rate of collision-induced dissociation gas Ar	0.3 mL/min
